# The Commencement Exercises of the Atlanta College of Physicians and Surgeons

**Published:** 1905-05

**Authors:** 


### THE COMMENCEMENT EXERCISES OF THE ATLANTA COLLEGE OF PHYSICIANS AND SURGEONS.

THE commencement exercises of the Atlanta College of Physicians and Surgeons were held in the Grand Opera House on April 3d. There were forty-eight graduates in medicine and thirty-six in pharmacy. Certificates of distinction were awarded to the following graduates: Jas. B. Baird, Jr., E. C. Crummey, G. U. McDonell, Y. A. Little and H. L. Simpson. The following appointments were announced: Grady Hospital—J. B. Baird, Jr., T. P. Longino, B. M. Kline; Presbyterian Hospital—Y. A. Little, Homer Boatwright; Tabernacle Infirmary—J. T. Floyd. The annual address was delivered by the distinguished Dr. Richard Douglas, of Nashville, Tenn., and may be found elsewhere in this journal.

The following is the annual report of the Dean, Dr. W. S. Kendrick:

"Mr. President and Gentlemen: It is with much pleasure that I report to you that the session just closed has been by far the most successful and satisfactory in the history of the institution. The attendance has been more than 16 per cent. larger than for last session. The student *esprit de corps* has been of a higher order than ever, and the actual work accomplished has been most gratifying to the faculty.

"We can not refrain from calling your especial attention to the high order of work being done in medical colleges now and to the superior class of men entering the profession now as compared with the work done and the men taking up the study of medicine even so late as ten years ago. In no other line ot education, professional or otherwise, have strides been made which are so little short of marvelous. The doctor of to-morrow will be a much better educated, a much more cultured, a much more rational and a correspondingly more powerful man than the doctor of yesterday or even of to-day. The profession no longer rests under the reproach of being a refuge for men who are fit for nothing else. Surviving the age of alchemy and the stigma of superstition and witchcraft, it has come into its rightful heritage—that of a truly learned profession. The healing art, once so occult, has become a science; and no profession so scientific has become practically humanitarian. We can but claim that in the evolution witnessed by the past fifteen years in medical education the institution over which you preside has had her full share.

"An epoch-making year confronts the school. During the past twelve months plans have been carefully laid to spend large sums in the erection of buildings. Our present main building was erected fifty years ago. It was well built and still furnishes ample room in connection with other buildings which we have erected, but is old, and it is proposed to replace it before the next session with another structure equipped in the most modern way. The college owns extensive grounds and plans are now being perfected to erect more satisfactory hospital quarters. Suffice it to say that no medical college anywhere in this section of the South is anything like so well equipped as this one will be.

"In attendance upon lectures in the three departments of medicine, pharmacy and dentistry there have been this year 501 students. Two hundred and fifty of these have been in the medical department and forty-eight ask at your hands the degree of doctor of medicine; 101 have attended the college of pharmacy and thirty-six are candidates for the degree of graduate in pharmacy; 150 have been enrolled in the dental department and of these a number will at a future date ask for the degree of doctor of dental surgery.

"Of the 250 medical students, 181 are from Georgia, 22 from Alabama, 12 from Florida, 9 from South Carolina, 7 from Texas, 5 from Mississippi, 2 from North Carolina, 2 from Kentucky, 2 from Tennessee, 2 from Cuba, 1 from Michigan, 1 from New York, 1 from Indian Territory, 1 from Louisiana and 1 from Canada.

"These have been classified as follows: First year, 62; second year, 55; third year 71; fourth year, 56; post-graduates, 6.

"The faculty takes great pleasure in commending to the public every young gentleman who will receive his diploma this evening. They have had opportunities of no mean degree, and their future attainments will doubtless reflect credit upon their alma mater

Respectfully submitted.

W. S. KENDRICK,
Dean of the Faculty."

The following is the list of graduates:

Section 1—John Edge Maines, Clyde Lattimore Carter, George Nowlan MacDonell, Louis Hollander, Louie Frank Grubbs, Francis Monroe Martin, Scott Mendenhall, Harold Marvin Babb, Joseph Oscar Kinard, Charles Davis Heard, Thomas Jonathan Jackson, Homer Boatwright, Russell Park Glenn, Edmond Franklin Saxon, John Henry Kelley, Elihu Posey Pruitt.

Section 2—John Howard Hall, Marion Sims Richardson, P. A. Tatum, Norman Wrightman McLeod, James Andrew Combs, George Malcolm Taylor, Luther Asbury DeLoach, Young Allen Little, Henry Thomas Simpson, Bascom Osborn Quillian, I. E. C. W. Smith, George Dawson Walker, Otis Hackett Johnson, Henry Jason Ault, Lovick Pierce Longino and Robert Howard Huntington.

Section 3.—William Nevin Adkins, John Thomas Floyd, Thomas Henry Jarman, Young Harris Yarbrough, Edward Pinson McLennan, Austin L. Smith, William Claybourne White, Bernard McHugh Cline, James Bozeman Baird, Jr., Walter Arthur Miller, Thomas Fletcher Harper, Everett Carlisle Major, Robert Grier Stephens, Alex Hamilton Beazley, Elias Cameron Crummey and Alvin J. Whelchel.

